# Biologically Active Compounds and Antioxidant and DNA-Protective Potential of Rhodope Avens *(Geum rhodopaeum* Stoj.&Stef.) Dry Tinctures

**DOI:** 10.3390/molecules31101643

**Published:** 2026-05-13

**Authors:** Ivanka Dimitrova-Dyulgerova, Silviya Mladenova, Elena Apostolova, Vasil Georgiev, Samir Naimov, Tsvetelina Mladenova, Ivayla Dincheva, Atanas Pavlov, Iliya Slavov, Rumen Mladenov

**Affiliations:** 1 Department of Botany and Biological Education, Faculty of Biology, University of Plovdiv “Paisii Hilendarski”, 4000 Plovdiv, Bulgaria; ivadim@uni-plovdiv.bg (I.D.-D.); cmladenova@uni-plovdiv.bg (T.M.); rummlad@uni-plovdiv.bg (R.M.); 2 Department of Human Anatomy and Physiology, Faculty of Biology, Plovdiv University “Paisii Hilendarski”, 4000 Plovdiv, Bulgaria; silviamladenova.sm@uni-plovdiv.bg; 3 Department of Molecular Biology, Faculty of Biology, University of Plovdiv “Paisii Hilendarski”, 4000 Plovdiv, Bulgaria; naimov0@uni-plovdiv.bg; 4 Laboratory of Cell Biosystems, Institute of Microbiology, Bulgarian Academy of Sciences, 139 Ruski Blvd., 4000 Plovdiv, Bulgaria; vasgeorgiev@microbio.bas.bg (V.G.); a_pavlov@uft-plovdiv.bg (A.P.); 5 Department of Medical Biochemistry, Faculty of Pharmacy, Medical University of Plovdiv, 15A Vasil Aprilov Blvd., 4002 Plovdiv, Bulgaria; 6 Department of Agrobiotechnologies, Agrobioinstitute, Agricultural Academy, 1164 Sofia, Bulgaria; ivadincheva@abi.bg; 7 Department of Analytical Chemistry and Physical Chemistry, Technological Faculty, University of Food Technologies, 4002 Plovdiv, Bulgaria; 8 Department of Biology, Faculty of Pharmacy, Medical University of Varna, 9002 Varna, Bulgaria; ijelev80@abv.bg

**Keywords:** *Geum rhodopaeum*, microscopic characteristics, HPLC phenolic profile, GC/MS identification, in vitro antioxidant activity, DNA protective assay

## Abstract

This article is the first report highlighting Rhodope avens as a natural source of bioactive compounds with potential pharmaceutical applications. Dry tinctures obtained from herba (HDT) and rhizoma (RDT) from the Balkan Peninsula endemic plant *Geum rhodopaeum* were analyzed and tested for antioxidant activity (DPPH, ABTS, FRAP, CUPRAC assays) and DNA nicking protection potential. The tinctures were characterized by a high total polyphenol content (total the polyphenols in RDT were more than twice that of HDT, i.e., 516.6 mg GAE/g dt; *p* < 0.01). HPLC analysis revealed the presence of 14 phenolics, with the main one being (over 3 mg/g dt) (+)-catechin (28.21 mg/g dt for RDT; 9.67 mg/g dt HDT), followed by protocatechuic acid, salicylic acid, and rutin. Rosmarinic acid content is herein first reported for the genus *Geum*. GC/MS analysis identified 41 compounds, among which monosaccharides predominated (with the highest fructose content), followed by gluconic acid, glucose-1-phosphate, mannitol, sorbitol, and glycerol in HDT, along with xylitol in RDT. The tinctures exhibited strong antioxidant potential as evaluated by the methods used, with the most pronounced effect found in the CUPRAC assay (5 458.87 μM TE/g dt for HDT; 10 073.99 μM TE/g dt for RDT; *p* < 0.01), as confirmed in comparison to proven antioxidants such as BHT and vitamin C. DNA-protective activity against oxidative damage was established and microscopic identification of the herbal substances was also performed.

## 1. Introduction

Nowadays, the importance of plants as a source of natural phytochemicals with valuable biological properties—such as antioxidant, antimicrobial, antitumor, and other activities—for the development of new drugs and therapies is increasingly recognized. Some species of the genus *Geum* L. (Rosaceae family) have attracted attention since ancient times due to their medicinal benefits and have been traditionally used in Europe and Asia to treat various inflammatory diseases [1,2,3,4]. Decoctions, prepared from *G. aleppicum*, *G. japonicum* and *G. rivale*, have been applied in East Asian traditional medicine as astringents, diuretics and antiseptic agents [5,6]. Infusions of *G. rivale* have also been employed for the treatment of fever, muscle pains, and inflammatory diseases of the gastrointestinal tract, urinary tract, skin, and others [7]. *G. urbanum* is a well known medicinal plant, traditionally used in various European countries to treat diarrhea, dysentery, gingivitis, hemorrhoids, leucorrhea, rheumatism, and gout [8,9,10,11]. Another *Geum* species (*G. iranicum*) is known for treating frostbite, in addition to being an astringent [12].

The current state of research of the *Geum species* indicates a growing interest in its bioactive compounds and potential pharmaceutical applications. The chemical composition of the most studied species (*G. urbanum*, *G.rivale*, *G. japonicum*) indicated the presence of tannins, flavonoids, phenolics, and triterpenoids in their extracts, as well as monoterpenoids and fatty acids in their essential oil compositions [1,2,3,11,13,14,15,16,17,18]. The seeds of *G. urbanum* and *G. rivale* are also mentioned as a valuable source of unsaturated fatty acids and active phenolics [10]. For the species *G. reptans*, *G. montanum*, *G. bulgaricum*, and *G. bulgaricum* × *G. reptans* (aerial parts), [19] report over 120 primary and secondary metabolites, including biologically active phenolic compounds such as gallic acid, caffeic acid, hydroxycinnamic acid, and others. A comprehensive review of the phytochemistry of the genus *Geum* to date summarizes more than 300 components isolated from the underground and aboveground parts of the studied species, namely: *G. urbanum*, *G.rivale*, *G. japonicum*, *G. reptans*, *G. montanum*, *G. aleppicum*, *G. bulgaricum*, *G. iranicum*, and *G. kokanicum* [4]. The phytochemicals are grouped as follows: terpenes (monoterpenes and sesquiterpenes in the composition of essential oils; diterpenes and triterpenes), phenylpropanoids (gein, eugenol, apiol, myristicin, etc.), tannins (gallotannins and ellagitannins), flavonoids, aromatics, steroids, and others (alcohols, aldehydes, alketones, carboxylic acids, alkanes and alkenes, etc.). Various pharmacological effects have been reported for *Geum* species, due to the bioactive phytochemicals identified in them. Different extracts from *G. urbanum* and *G. rivale* demonstrated significant antioxidant and antimicrobial activities [7,10,15,20,21,22]. Other studies also report the cardiogenic [23], anti-inflammatory [24], antidiabetic [25,26], antineoplastic, and antiviral properties [11] of *G. urbanum* extracts. A wide range of biological activities have been demonstrated for extracts and active fractions of *G. japonicum*: antihypertensive [27], neuroprotective [28], antidiabetic [26], angiogenic and cardiogenic [29], anticoagulant [30], and cardioprotective [18]. Alpha-glucosidase inhibitory potential has been reported for *G. aleppicum*, due to certain components such as gemin A and quercetin-3-O-glucuronide [31], as well as potent the anti-*Helicobacter pylori* effect of the *G. iranicum* tannin fraction [12]. A methanol extract from *G. iranicum* roots exhibits central analgesic as well as anti-inflammatory activities [3].

In light of the aforementioned evidence, our attention turned to another representative of the genus *Geum*, namely *Geum rhodopaeum* Stoj. & Stef. commonly known ‘Rhodope avens’. The species is rhizomatous perennial and grows primarily in temperate biomes in wet places, near streams, and 1200–1500 m a.s.l. The native range of *G. rhodopaeum* is the Balkan Peninsula—Bulgaria, Greece, and Serbia [32]. It is one of the eight *Geum* representatives of Bulgarian flora, found in the following floristic regions: The Rhodopes (West), Sredna Gora (West), Pirin (South), and Western Frontier Mountains [33]. The species is legally protected by the national Biological Diversity Act and has been assigned a national IUCN threat category of ‘Near-Threatened’ (NT) [34]. The endemic taxon has not been studied phytochemically and pharmacologically in Bulgaria to date. The scientific literature only contains data on the composition of essential oil obtained by hydrodistillation from air-dried aerial parts of *G. rhodopaeum* individuals growing in Serbia [35]. The authors report sixty-four compounds in the oil, with α-bisabolol as the major one.

To the best of our knowledge, there are no reports of phytochemical or biological studies on *G. rhodopaeum* extracts. Therefore, we aimed in the present work to determine the total phenolic content and some of the individual phytochemicals of dry ethanol extracts from the underground and aboveground parts of *G. rhodopaeum* by HPLC and GC-MS as well as to evaluate their antioxidant and DNA-protective potential. In addition, we perform a microscopic examination of the herbal substances.

## 2. Results and Discussion

### 2.1. Total Polyphenols and Main Phenolic Compounds in Geum rhodopaeum Tinctures

It was found that dry extracts from *G. rhodopaeum* had a high total polyphenolic content (TPC): 212.92 ± 22.47 mg GAE/g dt in the herba dry tincture (HDT) and 2.4 times more in the rhizome dry tincture (RDT), namely 516.60 ± 18.44 mg GAE/g dt (*p* < 0.01). The yield of HDT was 8.9768 g dt/100 g dry material, and the yield of RDT was 17.3799 g dt/100 g dry material, which indicates a more abundant amount of ethanol-soluble phytochemicals in the rhizomes—a finding supported by the measured TPC value.

HPLC analysis showed the presence of five flavonoids and nine phenolic acids in the extracts studied (Table 1, Figures S1 and S2).

In the HDT catechin had the highest concentration (9.67 mg/g dt), followed by rutin (5.58 mg/g dt) and salicylic acid (5.43 mg/g dt). In descending order, protocatechuic acid, rosmarinic acid, *p*-coumaric acid, and epicatechin were found in varying concentrations. The concentration of the other phenols was below 1%, and chlorogenic acid was not found. In the RDT, catechin was also found in the highest concentration (28.21 mg/g dt), followed by protocatechuic acid (17.62 mg/g dt) and salicylic acid (6.28 mg/g dt). In rank order, from 4 mg/g dt to 1 mg/g dt, epicatechin, rutin, vanillic, rosmarinic, *p*-coumaric, syringic, and gallic acid were found. The flavonols quercetin and kaempferol, as well as chlorogenic and ferulic acid, were present in small quantities (Table 1). In both extracts, the flavanone hesperidin was below the limit of quantification, and caffeic acid was not identified.

The total polyphenol content and HPLC phenolic profile revealed the presence of bioactive phenols in both extracts, with higher concentrations found in the rhizomes than in the aerial parts of the plant. This could be explained by the ability of the rhizomes to accumulate more tannins and phenolic acids, often found in many herbaceous medicinal plants of the Rosaceae family [8,10,16]. Similar results have been reported for *G. urbanum* and *G. rivale* [2,4,15,20,36]. The total polyphenol content (TPC) found in *G. rhodopaeum* extracts differs to some extent from the values reported for other *Geum* species. The *G. rhodopaeum* HDT contains 9.4% more total polyphenols than those found in the dry methanol extract (DME) of *G. rivale* and 11.7% more than those in the *G. urbanum* DME [20]. With regard to the underground parts, the authors report about 46% more polyphenol content in *G. rivale* (DME), and slightly less in *G. urbanum* (DME), compared to what we found for *G. rhodopaeum* (RDT). An extraction yield of 29.94% has been reported [22] for *G. urbanum* rhizomes (70% ethanol extract), with a measured TPC of 234.52 mg GAE/g extract, which is significantly lower than the value found for the *G. rhodopaeum* RDT (516.60 mg GAE/g dt). Studies on *G. aleppicum* (hydroethanolic extract of the aerial parts) indicate a significantly lower TPC (131.45 mg GAE/g dry extract) [37] compared to that of the *G. rhodopaeum* HDT. The observed differences in total phenolic content between the extracts obtained from the same plant parts using the same extraction solvent highlight the importance of species-specific characteristics and habitat conditions.

HPLC analysis of the *G. rhodopaeum* dry tinctures confirmed the presence of bioactive phenolic acids and flavonoids, previously identified in the genus *Geum*, namely rutin, quercetin, kaempferol, (+)-catechin, epicatechin, protocatechuic, salicylic, *p*-coumaric, gallic, vanillic, chlorogenic, and ferulic acids [1,4,11,15,16,17,19,37,38]. Caffeic acid was not detected, although it has been reported for *G. rivale* [2,39], *G. japonicum* [1], *G.aleppicum* [37], *G. urbanum* [11,17], *G. montanum*, G. *reptans*, G. *bulgaricum*, and G. *bulgaricum* × *G. reptans* [19]. Among the last-mentioned four species of the genus *Geum*, found in Bulgarian flora [19], gallic acid is the most abundant of the phenolic compounds, protocatechuic acid is present in small quantities, traces of salicylic acid have been detected, and of the flavonoids, only epicatechin has been identified (in methanolic extracts from aerial parts). By comparison, in our studies, (+)-catechin was among the most abundant phenols, along with protocatechuic acid, salicylic acid, and rutin, while gallic acid was significantly less represented. Similarly to our data, higher levels of (+)-catechin compared to epicatechin have been reported for *G. urbanum* underground parts [38] and *G.iranicum* [12]. Protocatechuic acid is also present in the hydroethanolic extract of *G. aleppicum* aerial parts, but in a quantity nearly 19 times lower (0.215 mg/g dry extract) compared to that found in the dry tincture of *G. rhodopaeum* aerial parts [37]. With regard to vanillic acid, there are also quantitative differences compared to other species—for example, in the aerial parts of *G. reptans*, it is found at 40.7 µg/g dw [19], whereas in our extract from the same part it is found at 0.57 mg/g dt, and in its underground parts it is found at more than 3 mg/g dt. Rosmarinic acid, which was present in our extracts at concentrations of 2–3 mg/g dt, has not been identified to date in *Geum* species. Chlorogenic acid, which was found in small amounts in the *G. rhodopaeum* rhizome extract and was absent in the aerial parts extract, was present in low concentrations in the aboveground parts (50% ethanolic extract) of *G. aleppicum* [37], and it prevails in the same plant part (methanolic extracts) of *G. urbanum* [38]. The solvent used and the method of preparing the extract also play a role in the quantitative differences.

Given the comparative analysis of flavonoid and phenolic acid content performed, it is evident that the phenolic profile of the endemic species *G. rhodopaeum* exhibits some qualitative and quantitative differences from other *Geum* species. The presence of high amounts of bioactive phenols suggests that its extracts have beneficial health effects. For example, catechins are known for their pronounced cardioprotective properties. Recent findings suggest that they also influence key cellular processes associated with cardiovascular disease [23]. In addition, catechins stimulate osteoblasts, inhibit bone resorption, and reduce the risk of fractures [40]. Another well represented flavonoid in the extracts, the flavonol glycoside rutin, is well known for its antioxidant, anti-inflammatory, cardioprotective, neuroprotective, antidiabetic, antimicrobial, and anticancer properties [41,42]. Rutin helps keep blood vessels flexible and has a beneficial effect on chronic venous insufficiency [41].

Protocatechuic acid, the main phenolic acid in the underground parts, exhibits a wide range of biological properties and plays a crucial role in prevention, largely due to its outstanding antioxidant activity [43]. Salicylic acid, which is also well represented in the tinctures, has pronounced anti-inflammatory, antipyretic, analgesic, antirheumatic, and anticoagulant properties [44,45]. Salicylic acid and its derivatives stimulate the regeneration of epithelial tissue and accelerate wound healing [46], and they are an ingredient in many cosmetic skincare products designed to treat acne, seborrheic dermatitis, psoriasis, and many others [47]. For rosmarinic acid, which we identified in the *G. rhodopaeum* extracts, significant therapeutic potential has been described. Its antioxidant, anti-inflammatory, antidiabetic, antiallergic, cardioprotective, antiangiogenic, antiviral, anticancer, and antimicrobial properties have been reported [48,49]. Ijaz et al. [50] report the pronounced hemoprotective and chemotherapeutic effects of rosmarinic acid and its derivatives. Rosmarinic acid is found in the families Lamiaceae and Boraginaceae [51,52], Apiaceae, Araliaceae, and Fabaceae [53,54], and sporadically in some others. In recent years, evidence has emerged of its presence in members of the Rosaceae family, such as in *Filipendula ulmaria* L. (4 mg/g dt) [55] and *Potentilla argentea* L. (10.83 mg/g dt) [56] dry ethanol tinctures. Rosmarinic acid might be a hemotaxonomic marker for *G. rhodopaeum*, given that it has not yet been detected in other *Geum* species.

### 2.2. Gas Chromatographic and Mass Spectrometric Analysis of G. rhodopaeum Tinctures

GC–MS analysis revealed 41 compounds in the analyzed extracts (Table 2, Figures S3 and S4), of which 20 were common to both samples. In the dry tincture from the aerial parts (HDT), 37 constituents were identified, representing 98.7% of the total composition. Among them, 17 were found in concentrations above 1%, and 9 exceeded 3%, with the main ones being two fructose isomers, gluconic acid, glucose-1-phosphate, mannitol, sorbitol, glycerol, mannose, and glucose.

The metabolite profile of HDT was dominated by primary metabolites. Sugars were the most abundant group, represented by monosaccharides (36.05% of all identified compounds), followed by organic acids and their derivatives (20.46%), sugar alcohols (19.87%), phosphorylated sugars (12.05%), glycerides (4.78%), fatty acids (4.04%), oxygenated diterpenes (1.33%), and amino acids (0.66%). Within the group of fatty acids, the omega-3 polyunsaturated linolenic acid was the predominant representative. In contrast, secondary metabolites (phenolic acids, cumarin) accounted for only 0.76% of the composition. In the dry tincture prepared from the underground parts (RDT), 25 components were identified, representing 98% of the total composition. Of these, 18 compounds were present at levels above 1%, while nine exceeded 3%. The major constituents included two fructose isomers, two glucose isomers, two galactose isomers, mannose, xylitol, and glucose 1-phosphate. Similarly to HDT, the volatile composition of RDT was largely dominated by primary metabolites, which accounted for 99.22% of the identified components. Sugars were the most abundant group (71.11%), followed by sugar alcohols (11.46%), organic acids and their derivatives (7.64%), sugar esters (5.70%), amino acids (2.39%), and fatty acids (0.92%). Among the secondary metabolites, only the alkane n-octacosane was present, accounting for less than 1% of all identified components.

The results of the GC-MS analysis can be compared with similar data on extracts from other *Geum* species. A single GC-MS analysis for *G. rhodopaeum* was conducted on the composition of essential oil obtained by hydrodistillation of the aerial parts, wherein the main components (over 5%) were: α-bisabolol, myrtenal, myrtanal, 1-isopropyl-4,8-dimethylspiro [4.5]dec-8-en-7-one, palmitic acid, and 1-octen-3-ol [35].

The primary metabolites reported to date for the extracts of *Geum* species include saccharides, organic acids, fatty acids, fatty alcohols, glycerides, phytosterols, tocoferols, and hydrocarbons in varying amounts, with saccharides being the most abundant [1,4,12,19,21]. Among the sugars, fructose and glucose account for the largest proportion in the Bulgarian populations of *G. reptans*, *G. montanum*, *G. bulgaricum*, and the hybrid *G. bulgaricum* × *G. reptans*, followed by sucrose and raffinose [19]. Sucrose has also been reported by other authors for *G. montanum* [4,21], as well as for *G. iranicum* [12]. The disaccharide vicianose has been identified in *G. urbanum* [1]. In *G. rhodopaeum*, monosaccharides (fructose, glucose, galactose, and mannose) were the main sugars, whereas vicianose and raffinose were not detected, and sucrose was present only in the RDT at a low concentration of 1%. In contrast, in *G. iranicum* it is the main sugar component (8.16% in the dried roots), probably responsible for the antibacterial effect against *Helicobacter pylori* [12]. For *G. reptans*, *G. montanum*, *G. bulgaricum*, and *G. bulgaricum* × *G. reptans*, the sugar alcohols include myo-inositol (the main one), erythritol, and arabinitol, while the main organic acids are malic, shicimic, and succinic acids [19]. By comparison, in *G. rhodopaeum* extracts we found varying amounts of mannitol, xylitol, sorbitol, and galactitol as well as gluconic, quinic, citric, and maleic organic acids, glutamic and pyroglutamic amino acids, and *n*-octacosane—compounds that have not previously been reported in the genus *Geum*. The previously reported glycerol, phytol, palmitic, linoleic, linolenic, and stearic fatty acids, as well as arabinitol and erythritol and malic, shicimic, and succinic acids [19,21], were also detected in *G. rhodopaeum*.

It can be assumed that the high content of the aforementioned primary metabolites in *G. rhodopaeum* extracts determines their nutritional and health benefits. Some of them are widely used as sweeteners and food additives, as well as in medicinal products, such as fructose, mannose, xylitol, sorbitol, mannitol, gluconic acid, glucose 1-phosphate, and glycerol [57,58,59,60,61,62].

### 2.3. Biological Activity Evaluation

#### 2.3.1. Antioxidant Activity

The high content of total polyphenols and the phenolic profile of *G. rhodopeum* tinctures, indicating the presence of proven antioxidants, was the basis for studying their antioxidant potential. Four in vitro assays were applied, the results of which are presented in Table 3. Based on the data in the table, it is clear that both extracts showed antioxidant activity, with the effect of the tincture from the underground parts (RDT) being more than twice as strong as that of the aboveground parts (HDT), as confirmed by all of the applied methods. Regarding the radical-scavenging ability of the tinctures, it was more pronounced for the DPPH radical (1371.77 μM TE/g dt for the HDT and 2903.76 μM TE/g dt for the RDT) compared to that for the ABTS radical. The power of the tinctures to reduce ferrous ions was similar in strength to that established in relation to DPPH radical scavenging. The capacity for reducing cupric ions was the most pronounced, with the highest value being expressed (5 458.87 μM TE/g dt for the HDT and 10 073.99 μM TE/g dt for the DRT). The data clearly demonstrated that the different phytochemical compositions of the HDT and RDT have a significant effect on their antioxidant properties. The RDT exhibited significantly higher contents of (+)-catechin (28.21 ± 0.83 mg/g dt vs. 9.67 ± 0.92 mg/g dt), (−)-epicatechin (4.03 ± 0.30 mg/g dt vs. 2.02 ± 0.34 mg/g dt), and protocatechuic acid (17.62 ± 0.73 mg/g dt vs. 4.06 ± 0.73 mg/g dt) compared to the HDT (Table 1). These antioxidants preferentially utilize the sequential proton-loss electron-transfer (SPLET) mechanism of antioxidant action, which is consistent with the significantly higher antioxidant activity of the RDT observed in the ABTS, FRAP, and CUPRAC assays compared to the HDT (Table 3). It is well known that (+)-catechin, (−)-epicatechin, and protocatechuic acid exhibit good bioavailability and possess great potential for the prevention of chronic diseases associated with oxidative stress.

Butylhydroxytoluene (BHT) and L-ascorbic acid were used as positive controls. The antioxidant activity of the rhizome dry tincture (RDT) was equal in strength to the BHT (a commonly used synthetic antioxidant) in terms of ABTS radical-scavenging activity, but in DPPH radical-scavenging activity, ferric reducing antioxidant power, and cupric reducing antioxidant capacity, it even exceeded it. The antioxidant power of the tinctures was confirmed when comparing the data with the other positive control (vitamin C), especially for the RDT, in which the ability to reduce cupric ions is even more pronounced.

Previous studies have also found that *Geum* species exhibit in vitro antioxidant properties in varying degrees. Aboveground and underground plant parts were studied using different solvents, methods for the preparation of extracts and fractions, and antioxidant activity assays. Aqueous ethanolic (70%) extracts of *G. urbanum* rhizomes demonstrated pronounced ability to scavenge ABTS cation radicals (2.97 mmol TE/g extract) and DPPH radicals (1.15 mmol TE/g extract), which are comparable with the radical-scavenging activities we determined for the *G*. *rhodopaeum* rhizome dry tincture. However, with regard to Fe-reducing potential, the measured values (0.39 mmol TE/g extract) are nearly eight times lower than those in our extracts [22]. The remarkable scavenging activity of an ethanolic extract of *G. urbanum* aerial and underground parts against DPPH radicals has also been mentioned [63]. Methanolic extracts from *G. urbanum* herba and roots and their fractions exhibit antiradical activity [15]. The authors found that the ethyl acetate fraction has the best ability to neutralize free radicals (DPPH and superoxide anion).

The antioxidant capacities of different methanolic extracts and fractions from *G. urbanum* and *G. rivale* aerial and underground parts have also been proven [20]. Significant correlations were found between TPC values and antioxidant activity, evaluated by DPPH, FRAP, and linoleic acid peroxidation assays. The authors report that ethyl acetate and *n*-butanol are proven to be the most efficient solvents to concentrate antioxidant compounds from methanolic extracts, and the rhizomes of *G. rivale* exhibit the highest antioxidant potential. The aqueous and ethyl acetate fractions of a total ethanolic (70%] extract of *G. rivale* aerial parts also demonstrate high capacities for free-radical-scavenging activity (DPPH, ABTS, and superoxide anion radicals) comparable to that of the isolated individual components [7]. Another study [21] investigated the antioxidant capacity of an aqueous methanol (80%) extract of *G. urbanum* aerial parts, its fractions, and its isolated compounds using DPPH (2,2,1-diphenyl-1-picrylhydrazyl) and hydroxyl radical-scavenging assays, as well as a CUPRAC (cupric ion reducing antioxidant capacity) assay. The ethyl acetate fraction and isolated catechin and gemin A demonstrated high antioxidant properties. The high catechin content in our extracts (especially in the underground parts) may explain the strong antioxidant activity demonstrated in the four assays applied.

The extracts obtained from other *Geum* species are also effective for neutralizing free radicals, such as the *G. iranicum* root methanolic extract and its ethyl acetate fraction [26], and the *G. quellyon* sweet root methanolic extract with tannin content [64]. Other authors [37] report that the hydroethanolic extract from the aerial parts of *G. aleppicum* has strong DPPH radical-scavenging activity and ferric reducing antioxidant power (FRAP). Ellagitannins isolated from *G. japonicum* var. *chinense* demonstrate stronger oxygen radical antioxidant capacity (ORAC assay) compared to that exhibited by vitamin C [65]. A similar effect was found for the rhizome dry tincture (RDT) of *G. rhodopaeum*, in terms of its antioxidant capacity to reduce copper ions.

The comparative analysis of our data with those of other authors suggests that *G. rhodopaeum* exhibits strong antioxidant potential comparable to that of well-established medicinal *Geum* species.

#### 2.3.2. DNA-Protective Capacity

**Figure 1:**
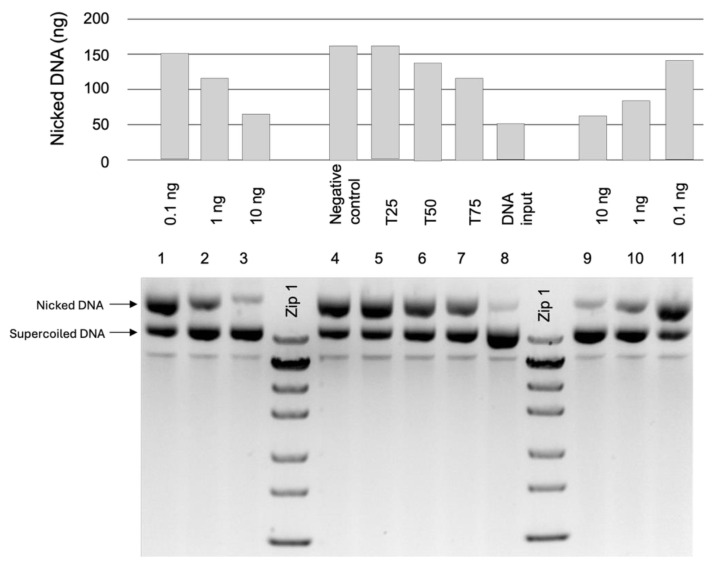
DNA protection assay with 1.2% agarose gel electrophoresis (bottom panel), and relative concentration (top panel) of necked plasmid DNA. 1–3—*G. rhodopaeum* HDT at concentrations of 0.1 1.0, and 10 ng/mL; 9–11—*G. rhodopaeum* RDT at concentrations of 10, 1.0, and 0.1 ng/mL; 4-Negative (water) control; 8—input DNA 5, 6, and 7; T25—Trolox 25 μg/mL; T50—Trolox 50 μg/mL; T75—Trolox 75 μg/mL; Zip1-Zip Ruler 1 (Thermo Scientific, SM1373, Waltham, MA, USA).

To determine the protective capacity of the *G. rhodopaeum* tinctures (HDT and RDT), serial dilutions of each extract were examined with an in vitro DNA nicking assay. Both tinctures were tested in three different concentrations—0.1, 1.0, and 10 ng/mL (Figure 1). In both cases, an inverse correlation between extract amount and DNA nicking was found. The DNA-protective effect was similar for both preparations. Ten nanograms of the extracts were sufficient for complete DNA nicking prevention. The amount of the nicked DNA was the same as the amount found in the input plasmid DNA and lower than the amount of nicked DNA in the 75 µg/mL Trolox control sample.

In the present study, the antioxidant activity of two plant extracts was evaluated using an in vitro DNA nicking assay, frequently used to study the ability of different compounds to prevent oxidative DNA damage. The results demonstrate that both extracts show a clear protective effect against nonspecific DNA cleavage. A dose-dependent protective effect was found for both extracts. Increasing the concentration of the extracts from 0.1 to 10 ng/mL resulted in a progressive reduction in DNA nicking. This finding indicates that the compounds present in the extracts effectively counteract the reactive oxygen species, formed by Fenton reagent, responsible for DNA strand breaks. Similar concentration-dependent antioxidant effects have been reported for numerous plant-derived extracts rich in polyphenolic compounds [55,66,67]. Methanolic extracts of *Geum quellyon*, which contain tannins, exhibit the ability to protect plasmid DNA from cleavage induced by hydroxyl radicals (*OH) and nitric oxide (NO) [64].

In our case, both plant extracts exhibited comparable levels of DNA protection, suggesting that their antioxidant capacities are relatively similar under the experimental conditions used. At the highest tested concentration (10 ng/mL), both extracts almost completely prevented DNA nicking, as demonstrated by the preservation of the intact plasmid DNA form. The identified compounds, phenolic acids and flavonoids, particularly protocatechuic acid, (+)-catechin, and (−)-epicatechin, may contribute to the prevention of DNA damage through their antioxidant and reactive oxygen species scavenging activities reported in the literature [43].

### 2.4. Microscopic Identification of Geum rhodopaeum Herbal Drugs

**Figure 2:**
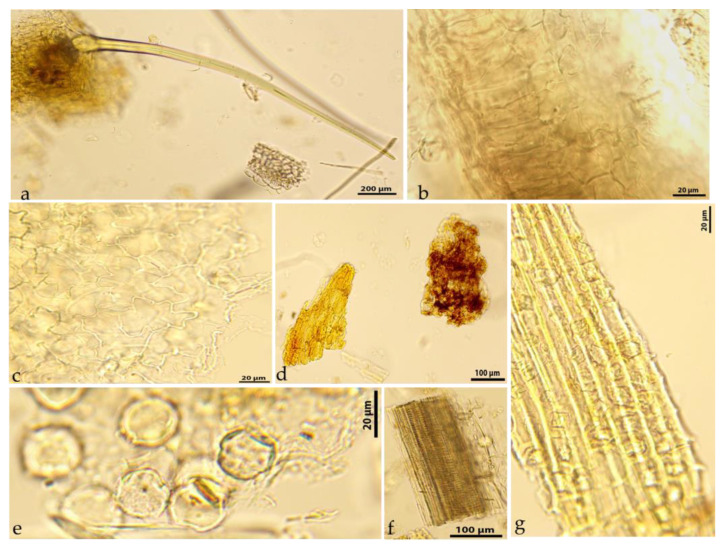
Microphotos of powdered *G. rhodopaeum herba*: (**a**)—straight unicellular covering trichomes and leaf lamina fragments; (**b**)—a close-up view of a bifacial leaf lamina in cross-section; (**c**)—the lower leaf epidermis with stomata; (**d**)—petal fragments; (**e**)—pollen grains; (**f**)—a vascular bundle fragment; (**g**)—parenchyma tissue with calcium oxalate drusen.

The microscopic analysis of powdered *Geum rhodopaeum herba* (Figure 2) revealed the following important diagnostic features, namely: numerous straight unicellular covering trichomes, varying from 300 to 1200 μm in length—alone or with parts of a leaf blade; leaf lamina fragments in cross-section (bifacial leaf structures); fragments of leaf epidermal tissue with anomocytic-type stomata and basal cells with curved anticlinal walls; orange-tinged pieces of petals; fragments of vascular bundles; tricolporate pollen grains, medium in size (about 25–30 μm in diameter), triangular to circular (from polar view) in shape, with the exine having sriate ornamentation with microperforations; and parenchyma tissue fragments with calcium oxalate crystals.

**Figure 3:**
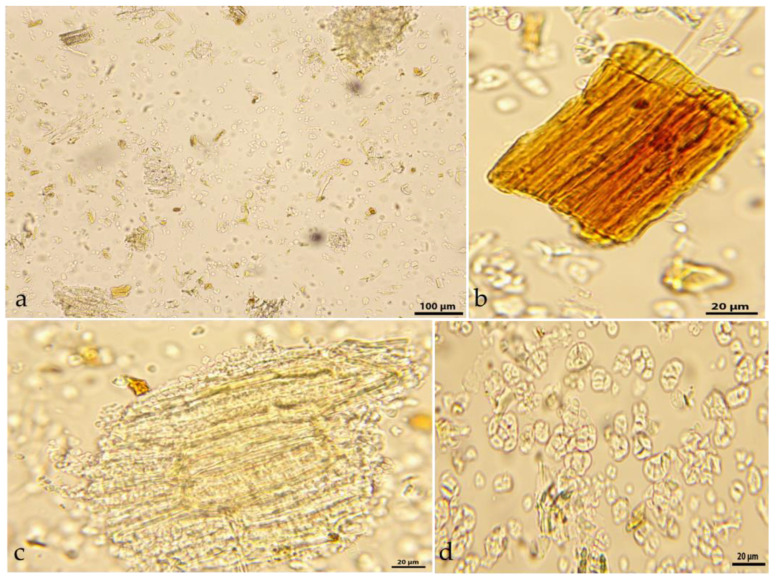
Microphotos of *G. rhodopaeum rhizoma* powdered herbal drug: (**a**)—typical microscopic features under low magnification; (**b**)—group of cells with catechins; (**c**)—starchy parenchyma tissue fragment; (**d**)—starch grains.

The powdered *G. rhodopaeum rhizoma* (Figure 3) showed the following important diagnostic microscopic features: numerous scattered starch grains with a diameter of 12–20 μm, spherical-to-oval shape; parts of starch-bearing parenchyma tissue; red-stained cells, containing catechins; and fragments of vascular bundles.

The microscopic evaluation of powdered HDT and RDT could provide basic information regarding the quality of the plant material, while the presence of colored groups of catechin-containing cells could serve as a marker of catechin-rich biomass with a high content of antioxidants and valuable pharmaceutical properties.

## 3. Materials and Methods

### 3.1. Collection of Plant Material and Preparation of Dry Tinctures

**Figure 4:**
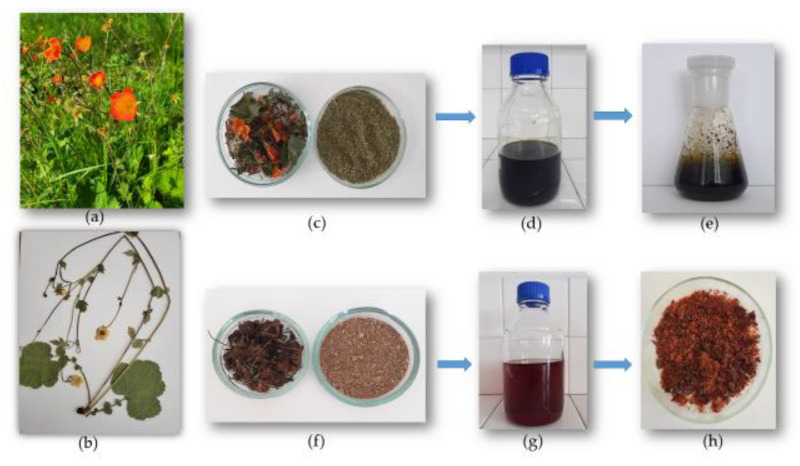
*Geum rhodopaeum* in its natural habitat (author’s photograph) (**a**), voucher specimen (**b**), and the main steps in the process of obtaining the tinctures: air-dried cut and powdered aboveground parts (*G. rhodopaeum herba*) (**c**); filtered tincture before vacuum drying (**d**); herba dry tincture—HDT (**e**); cut and powdered rhizomes (*G. rhodopaeum rhizoma*) (**f**); filtered rhizome tincture before vacuum drying (**g**); and rhizome dry tincture—RDT (**h**).

The plant material was collected in November 2024 (rhizome et radix, in the following text summarized as rhizome) and June 2025 (aerial flowering parts—herba) from Bulgaria, in the area of the Buynovo village, Western Rhodope Mountains (1358 m above sea level), with permission from the Ministry of Environment and Water in Bulgaria (permit No. 1066/ 27.11.2024). The species was identified at the Department of Botany, University of Plovdiv ‘Paisii Hilendarski’, Plovdiv, Bulgaria, and a voucher specimen (No. 063641) was deposited at the Herbarium of the Agricultural University in Plovdiv, Bulgaria—Herbarium code SOA. The collected plant material was dried in the shade at room temperature and then chopped and powdered using an electric laboratory grinder (GRINDOMIX GM 200). For the preparation of the tinctures, we followed the European Pharmacopoeia methodology by soaking one part of the plant material in ten parts of pure 96% ethanol for a 30-day period [68]. At the end of this period, the tinctures were filtered through an 8–12 μm pore-size filter and vacuum-dried with a BUCHI R-300 Rotavapor at 50 °C and 97 mbar. The extracted dry tinctures, the subject of the present study, differed visually in color, consistency and odor (Figure 4). The herba dry tincture (HDT) was dark green and oily, while the rhizome dry tincture (RDT) was red-brown, tannic and crumbly, with a pleasant sweet fragrance.

### 3.2. Determination of Total Polyphenols and Main Phenolic Compounds (HPLC Analysis)

The total phenolic content of the tinctures was analyzed by using the Folin–Ciocalteu method as reported previously [69]. Briefly, in a 96-well plate, 180 µL of Folin–Ciocalteu reagent was mixed with 20 µL of diluted tincture (in methanol, 1:100 *w*/*v*}. The plate was mixed for 2 min and then 100 µL of 7.5% sodium carbonate in water was added. The plate was incubated at 37 °C with mixing for 8 min, and the change in absorbance (λ = 750 nm) was measured (Multiskan FC, Thermo Fisher Scientific, Waltham, MA, USA) against a blank developed with methanol instead of tincture. The results were expressed as Gallic acid equivalents per gram dry tincture by using a standard curve.

High-performance liquid chromatography (HPLC) was used for the quantitative analyses of the phenolic acids and flavonoids in the tinctures as described previously [69]. Twenty milligrams of the tinctures was dissolved in 2 mL methanol, filtered by 0.45 µm CA syringe filters (Thermo Fisher Scientific, Waltham, MA, USA), and 20 µL was loaded for analysis. The HPLC system used included a Waters 1525 HPLC pump and a Waters 2484 dual λ Absorbance Detector (Waters, Milford, MA, USA). The separation was performed on a 5 µm, 25 cm × 4.6 mm Supelco Discovery HS C18 column (Merck KGaA, Darmstadt, Germany) with a flow rate of 1.0 mL/min by using a gradient mix of 1% acetic acid in water (Solvent A) and methanol (Solvent B) as described in [69]. The identification of the compounds was done by retention time. Standard curves, build with gallic, protocatechuic, vanillic, syringic, *p*-coumaric, and salicylic acids, as well as (+)-catechin, (−)-epicatechin, and hesperidin (detected at λ = 280 nm), and caffeic, ferulic, rosmarinic and chlorogenic acids, as well as rutin, kaempferol and quercetin (detected at λ = 360 nm) were used to quantify the compounds.

### 3.3. Gas Chromatography/Mass Spectrometry (GC/MS) Analysis

A portion of 5 mg of the dried extract was subjected to derivatization. The sample was first treated with 300 µL of methoxyamine hydrochloride solution (20 mg mL^−1^ in pyridine) and incubated in a thermoshaker at 90 °C with agitation at 300 rpm for 1 h. Subsequently, 100 µL of N, O-bis(trimethylsilyl)trifluoroacetamide (BSTFA) was added to the reaction mixture, followed by a second incubation at 75 °C and 300 rpm for an additional 1 h to complete the derivatization process. After the reaction, 1 µL of the final derivatized solution was injected into the GC–MS system for analysis.

Metabolite profiling was carried out using gas chromatography–mass spectrometry (GC–MS) on a 7890A gas chromatograph coupled to a 5975C mass-selective detector (Agilent Technologies, Santa Clara, CA, USA). Separation was achieved using an HP-5 ms capillary column (30 m × 0.32 mm, film thickness 0.25 μm) coated with dimethylsiloxane as the stationary phase. The oven temperature program started at 60 °C, followed by a gradual increase to 300 °C at a rate of 5 °C min^−1^, with a final hold of 10 min. Helium served as the carrier gas at a constant flow rate of 1.0 mL min^−1^. The injector and detector temperatures were both maintained at 250 °C. A 1 μL sample volume was injected using a split ratio of 10:1. Mass spectra were recorded within the scan range of 50–550 m/z. Compound identification was performed using AMDIS version 2.73 (NIST, Gaithersburg, MD, USA). The obtained spectra were compared with reference data from the Golm Metabolome Database [70] and the NIST Mass Spectral Library [71]. Retention indices (RIs) were calculated using a standard *n*-alkane calibration mixture (C8–C36; Restek, Teknokroma, Barcelona, Spain). Each kernel variety was analyzed in three independent replicates.

### 3.4. Determination of Biological Activities

#### 3.4.1. Antioxidant Activity Assays

Ten milligrams of the investigated tinctures were dissolved in 1 mL methanol and filtered by 0.45 µm CA syringe filters (Thermo Fisher Scientific, Waltham, MA, USA) to prepare the stock solutions. For the analyses of the antioxidant activities, serial dilutions (50-, 100- and 150-fold) of stocks were prepared and used in experiments. All reactions and measurements were performed by using a Multiskan FC microplate spectrophotometer (Thermo Fisher Scientific, Waltham, MA, USA). The results from all used methods were expressed as micromoles Trolox equivalents per gram dry tincture. Ascorbic acid and butylhydroxytoluene (BHT) were used as positive controls.

DPPH Assay: The abilities of tinctures to scavenge the DPPH radical were evaluated by using a previously described procedure [69]. In brief, 20 µL of diluted tincture were mixed with 280 µL of 0.1 mM DPPH in methanol, incubated for 15 min at 37 °C, followed by measurement of absorbance at λ = 515 nm. A negative control with methanol instead of the tincture was developed as well. The percentage of DPPH radical inhibition was calculated and used to determine the antioxidant activity of the tincture by using a calibration curve built by plotting the %DPPH inhibition vs. Trolox concentration.

ABTS assay: The abilities of the tinctures to scavenge the ABTS radical were evaluated by using a previously described procedure [69]. Briefly, 20 µL of diluted tincture was mixed with 280 µL of freshly prepared ABTS radical diluted in methanol to reach an absorbance of 0.9 at λ = 734 nm. The reaction was run for 15 min at 37 °C, and the absorbance at λ = 734 nm of tincture and a negative control (developed with methanol instead of the tincture) was recorded. The percentage of ABTS radical inhibition was calculated and used to determine the antioxidant activity of the tincture by using a calibration curve built by plotting the %ABTS inhibition vs. Trolox concentration.

Ferric reducing antioxidant power (FRAP) assay: The abilities of tinctures to reduce ferric ions were evaluated by using a previously described procedure [69]. In brief, 280 µL freshly prepared FRAP reagent (300 mM sodium acetate buffer to 10 mM 2,4,6 tripyridyl-s-triazine to 20 mM iron (III) chloride in ratios 10/1/1 *v*/*v*/*v*) was mixed with 20 µL of diluted tincture. The mix was incubated for 15 min at 37 °C, and the absorbance at λ = 593 nm was measured against a blank developed with methanol instead of the tincture. The antioxidant activity was calculated by using a calibration curve built by plotting the absorbance (λ = 593 nm) vs. Trolox concentration.

Cupric ion reducing antioxidant capacity (CUPRAC) assay: The abilities of the tinctures to reduce Cu (II) ions were evaluated by using a previously described procedure [69]. The reaction mixture was prepared by mixing 20 µL of diluted tincture, 70 µL ammonium acetate buffer, 70 µL copper dichloride hydrate, 70 µL neocuproine and 70 µL distilled water. The plate was incubated for 10 min at 37 °C, and the absorbance at λ = 450 nm was measured against a blank developed with methanol instead of the tincture. The antioxidant activity was calculated by using a calibration curve built by plotting the absorbance (λ = 450 nm) vs. Trolox concentration.

#### 3.4.2. DNA-Protective Activity Assay

The DNA-protective effect of the extracts was assessed using pGlO plasmid DNA (Bio-Rad Laboratories, Hercules, CA, USA) purified from *E. coli* strain Neb10 according to Rajiv et al. [72] and Andonova et al. [73]. Plasmid DNA was purified using QIAprep Spin Miniprep Kit (Qiagen, Hilden, Germany). Serial dilutions of extracts, starting from 0.1 ng/mL up to 10.0 ng/mL, were tested. As positives, negative controls of different concentrations (25, 50, and 75 μg/mL) of 6-Hydroxy-2,5,7,8-tetramethylchromane-2-carboxylic acid (Trolox, Sigma, Kawasaki, Japan) and demineralized water were used. The reactions were incubated at 37 °C for 30 min and the resulting products were separated with 1.2% agarose gel electrophoresis in 0.5× TBE buffer at 120 V for 2 h. The amount of nicked DNA was quantified using GelAnalyzer 23.1.1 (available at www.gelanalyzer.com by Istvan Lazar Jr., PhD and Istvan Lazar Sr., PhD, CSc.) [74].

### 3.5. Light Microscopy Assay of Herbal Drugs

The powdered aerial flowering parts (*G. rhodopaeum herba*) and underground parts (*G. rhodopaeum rhizoma*) were sieved through a 0.4 mm pharmacopeial sieve and analyzed microscopically to determine their diagnostic micro-features. The powdered herbal drugs were treated with a chloral hydrate solution (under heating), according to European pharmacopeial methodology [75]. A microscopic assay was carried out with a Magnum T CETI trinocular microscope, and microphotographs were taken with a Si 5000 5Mpx camera (Diagnostic Instruments, Inc., MI, USA).

### 3.6. Statistical Methods

The results obtained from the chromatographic and biological analyses are presented as mean values with corresponding standard deviations. Data processing was performed using Microsoft Excel (Microsoft Office 2003). Statistical significance was assessed by one-way analysis of variance (ANOVA), followed by Tukey’s post hoc test, using Statistics Kingdom [76].

## 4. Conclusions

The present study is the first report on the bioactive compounds in dry tinctures from the Balkan endemic plant *Geum rhodopaeum*, concerning their antioxidant and DNA-protective capacity. The findings revealed the presence of 55 phytochemicals, including phenols, carbohydrates, organic acids and their derivatives, sugar alcohols, sugar esters, glycerides, fatty acids and their esters, polyenes, amino acids, and hydrocarbons. The high total polyphenol content, including proven antioxidants such as (+)-catechin, rutin, protocatechuic acid, and salicylic acid, correlates with the significant antioxidant and DNA-protective activities demonstrated by the tinctures. In addition, the diagnostic microscopic characteristics of the plant substances (*G. rhodopaeum herba* and *G. rhodopaeum rhizoma*) were first described.

In brief, *G. rhodopaeum* demonstrates significant potential as a source of bioactive compounds with prospective applications in functional foods and pharmaceutical products, especially its underground parts. Further studies are needed in order to examine the other pharmacological properties of its extracts, as well as their safety. Due to its conservation status, research should also focus on the cultivation of this obviously valuable plant species.

## Figures and Tables

**Table 1:** HPLC quantification of some representative phenolics in *G. rhodopaeum* tinctures.

No.	Compounds	Content, mg/g dt (Mean ± SD) *	
		HDT	RDT
Flavonoids			
1	Rutin	5.58 ^b,^** ± 0.17	3.12 ^e^ ± 0.11
2	Quercetin	0.02 ^e^ ± 0.00	0.02 ^g^ ± 0.00
3	Kaempferol	0.37 ^e^ ± 0.02	0.38 ^g^ ± 0.01
4	(+)-Catechin	9.67 ^a^ ± 0.92	28.21 ^a^ ± 0.82
5	(−)-Epicatechin	2.02 ^d^ ± 0.34	4.03 ^d,e^ ± 0.30
Phenolic acids			
6	Gallic acid	0.75 ^e^ ± 0.04	1.19 ^f,g^ ± 0.09
7	Protocatechuic acid	4.06 ^c^ ± 0.73	17.62 ^b^ ± 0.73
8	Vanillic acid	0.57 ^e^ ± 0.11	3.20 ^d,e^ ± 0.07
9	Syringic acid	0.52 ^e^ ± 0.16	1.38 ^f,g^ ± 0.05
10	*p*-Coumaric acid	1.89 ^d^ ± 0.21	1.63 ^f^ ± 0.36
11	Salicylic acid	5.43 ^b^ ± 0.20	6.28 ^c^ ± 0.56
12	Chlorogenic acid	NF	0.43 ^f,g^ ± 0.01
13	Ferulic acid	0.29 ^e^ ± 0.01	0.33 ^g^ ± 0.01
14	Rosmarinic acid	2.40 ^d^ ± 0.03	2.82 ^d,e^ ± 0.02

HDT—Herba dry tincture; RDT—rhizome dry tincture; NF—not found. * The data, derived from three replicates, are shown as means ± standard deviation in mg/g dt (dry tincture). ** Means denoted by different small superscript letters are significantly different as per Tukey’s test (*p* < 0.01).

**Table 2:** Chemical composition (by GC/MS) of *G. rhodopaeum* dry tinctures.

Peak	RT	RI	Compound	Content (% of TIC) ^1^	
				HDT	RDT
1	5.53	1264	Glycerol	4.72 ^f^ ± 0.15	-
2	5.80	1296	Maleic acid	0.17 ^j^ ± 0.05	-
3	5.96	1307	Succinic acid	1.05 ^i,j^ ± 0.03	-
4	7.18	1455	Coumarin	0.31 ^j^ ± 0.02	-
5	7.54	1478	Malic acid	0.69 ^i,j^ ± 0.10	0.15 ^i^ ± 0.03
6	7.70	1494	Erythritol	0.21 ^j^ ± 0.03	-
7	7.80	1511	Salicylic acid	0.24 ^j^ ± 0.05	-
8	7.89	1517	Pyroglutamic acid	0.12 ^j^ ± 0.01	1.41 ^h^ ± 0.01
9	8.11	1549	Cinnamic acid	0.20 ^j^ ± 0.06	-
10	8.40	1609	Glutamic acid	0.53 ^j^ ± 0.06	0.93 ^h,i^ ± 0.12
11	8.53	1633	Xylose isomer	0.22 ^j^ ± 0.08	0.38 ^i^ ± 0.05
12	9.05	1644	Xylose isomer	0.21 ^j^ ± 0.09	0.92 ^h,i^ ± 0.11
13	9.91	1690	Xylitol	0.55 ^j^ ± 0.10	7.20 ^c^ ± 0.36
14	9.99	1703	Arabitol	0.29 ^j^ ± 0.03	2.27 ^g^ ± 0.13
15	10.46	1793	Shikimic acid	1.06 ^i,j^ ± 0.16	-
16	10.74	1804	Citric acid	0.16 ^j^ ± 0.04	1.58 ^g,h^ ± 0.12
17	10.95	1843	Quinic acid	0.88 ^i,j^ ± 0.12	2.83 ^f,g^ ± 0.05
18	11.26	1855	Fructose isomer	10.07 ^c,d^ ± 0.27	24.97 ^a^ ± 0.68
19	11.41	1864	Fructose isomer	10.93 ^c^ ± 0.64	6.27 ^d^ ± 0.25
20	11.48	1870	Mannose isomer	6.10 ^e^ ± 0.53	3.42 ^f^ ± 0.17
21	11.55	1875	Galactose isomer	1.25 ^i^ ± 0.06	7.31 ^c^ ± 0.24
22	11.59	1881	Glucose isomer	1.83 ^h,i^ ± 0.05	7.25 ^c^ ± 0.18
23	11.91	1883	Gluconate lactone	1.09 ^i,j^ ± 0.10	-
24	11.98	1887	Mannose isomer	0.74 ^i,j^ ± 0.06	0.15 ^i^ ± 0.01
25	12.09	1898	Galactose isomer	0.95 ^i,j^ ± 0.03	7.54 ^c^ ± 0.19
26	12.55	1901	Glucose isomer	3.28 ^g^ ± 0.05	10.44 ^b^ ± 0.28
27	12.62	1914	Mannitol	9.79 ^d^ ± 0.21	-
28	12.71	1921	Glucitol = Sorbitol	6.35 ^e^ ± 0.17	1.76 ^g,h^ ± 0.05
29	12.86	1926	Galactitol	2.42 ^h^ ± 0.10	-
30	13.25	1937	Glucose 1-phosphate	11.89 ^b^ ± 0.23	4.50 ^e^ ± 0.11
31	14.03	1985	Gluconic acid	15.10 ^a^ ± 0.34	1.79 ^g,h^ ± 0.02
32	15.15	2046	Palmitic acid	0.95 ^i,j^ ± 0.08	-
33	16.35	2083	Ethyl linolate	-	0.90 ^h,i^ ± 0.09
34	17.31	2112	Phytol	1.31 ^i^ ± 0.13	-
35	17.94	2208	Linoleic acid	0.35 ^j^ ± 0.08	-
36	18.06	2217	Linolenic acid	1.98 ^h,i^ ± 0.12	-
37	18.56	2241	Stearic acid	0.71 ^i,j^ ± 0.05	-
38	19.23	2313	Mannose-6-phosphate	-	1.09 ^h,i^ ± 0.08
39	21.68	2440	Gluconic acid-6-phosphate	-	1.14 ^h^ ± 0.18
40	23.85	2635	Sucrose	-	1.04 ^h,i^ ± 0.03
41	25.83	2799	*n*-Octacosane	-	0.76 ^h,i^ ± 0.06
	Total identified compounds, %			98.70	98.00

RT—Retention time; RI—retention (Kovat’s) index; TIC—total ion current; HDT—herba dry tincture; RDT—rhizome dry tincture. ^1^ Mean values ± standard deviation (*n* = 3). The small letters in superscript indicate the statistical differences in the column (*p* < 0.01; Tukey’s test).

**Table 3:** In vitro antioxidant activity of *Geum rhodopaeum* tinctures (μM TE/g dt).

Sample	ABTS Mean ± SD *	DPPH Mean ± SD	FRAP Mean ± SD	CUPRAC Mean ± SD
HDT	304.70 ^c,^** ± 50.25	1371.77 ^c^ ± 216.28	1037.09 ^d^ ± 141.96	5458.87 ^c^ ± 699.85
RDT	1170.29 ^b^ ± 175.40	2903.76 ^b^ ± 488.65	2995.95 ^b^ ± 321.72	10,073.99 ^a^ ± 1512.98
BHT	1299.34 ^b^ ± 172.31	1721.22 ^c^ ± 184.17	1528.73 ^c^ ± 141.01	3654.54 ^d^ ± 321.31
L-Ascorbic acid	3456.37 ^a^ ± 142.22	6009.05 ^a^ ± 322.96	5096.68 ^a^ ± 184.44	7698.91 ^b^ ± 222.64

* Mean values with standard deviations from eight independent reactions are presented. ** Means with different small letters in superscript in each column are significantly different according to one-way ANOVA with Tukey’s post hoc test (*p* < 0.01). ABTS, DPPH—radical-scavenging assays; FRAP—ferric reducing antioxidant power assay; CUPRAC—cupric reducing antioxidant capacity assay; HDT—herba dry tincture; RDT—rhizome dry tincture; BHT—butylhydroxytoluene; μM TE/g dt—micromoles of Trolox equivalents per gram of dry tincture.

## Data Availability

The original contributions presented in this study are included in the article/Supplementary Material. Further inquiries can be directed to the corresponding author.

## Supplementary Materials

The following supporting information can be downloaded at https://www.mdpi.com/article/10.3390/molecules31101643/s1, Figure S1: Typical HPLC chromatograms (280 nm and 360 nm) of the phenolic acids and flavonoids in *G. rhodopaeum* HDT: 1—Gallic acid, 2—Protocatehuic acid, 3—(+)-Catechin, 4—Vanillic acid, 5—Syringic acid, 6—(−)-Epicatechin, 7—*p*-Coumaric acid, 8—Salicylic acid, 9—Hesperidin, 10—Ferulic acid, 11—Rutin, 12—Rosmarinic acid, 13—Quercetin, 14—Kaempherol; Figure S2: Typical HPLC chromatograms (280 nm and 360 nm) of the phenolic acids and flavonoids in *G. rhodopaeum* RDT: 1—Gallic acid, 2—Protocatehuic acid, 3—(+)-Catechin, 4—Vanillic acid, 5—Syringic acid, 6—(−)-Epicatechin, 7—*p*-Coumaric acid, 8—Salicylic acid, 9—Hesperidin, 10—Chlorogenic acid, 11—Ferulic acid, 12—Rutin, 13—Rosmarinic acid, 14—Quercetin, 15—Kaempherol; Figure S3: GC/MS chromatogram of the volatile compounds in *G. rhodopaeum* HDT (main compounds over 3%): 1—Glycerol; 2,3—Fructose isomers; 4—Mannose; 5—Glucose; 6—Mannitol; 7—Glucitol = Sorbitol; 8—Glucose 1– phosphate; 9—Gluconic acid; Figure S4: GC/MS chromatogram of the volatile compounds in *G. rhodopaeum* RDT (main compounds over 3%): 1—Xylitol; 2,3—Fructose isomers; 4—Mannose isomer; 5—Galatose isomer; 6—Glucose isomer; 7—Galactose isomer; 8—Glucose isomer; 9—Glucose-1-phosphate.
