# Supplementary figures and images for: Biologically Active Compounds and Antioxidant and DNA-Protective Potential of Rhodope Avens (Geum rhodopaeum Stoj.&Stef.) Dry Tinctures
Source: Molecules. 2026 May 13;31(10):1643. doi: 10.3390/molecules31101643 (PMC13209557; doi:10.3390/molecules31101643)

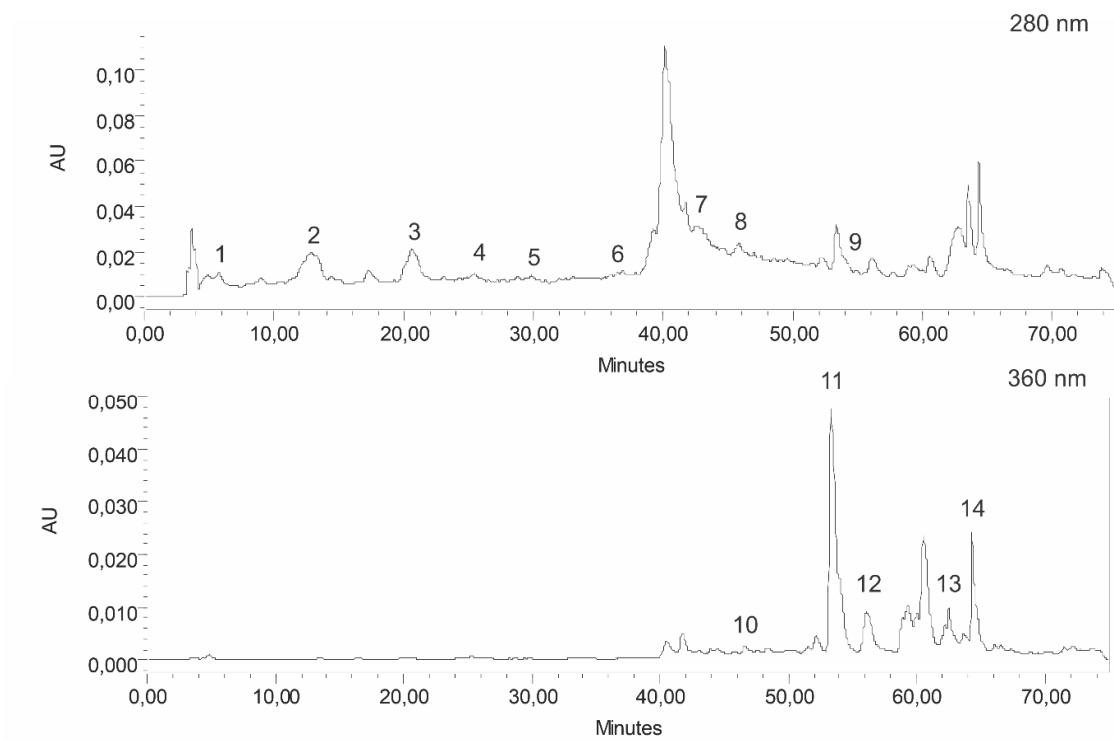

Supplement: Supplementary file 1 [file molecules-31-01643-s001.zip › Figures S1.pdf]

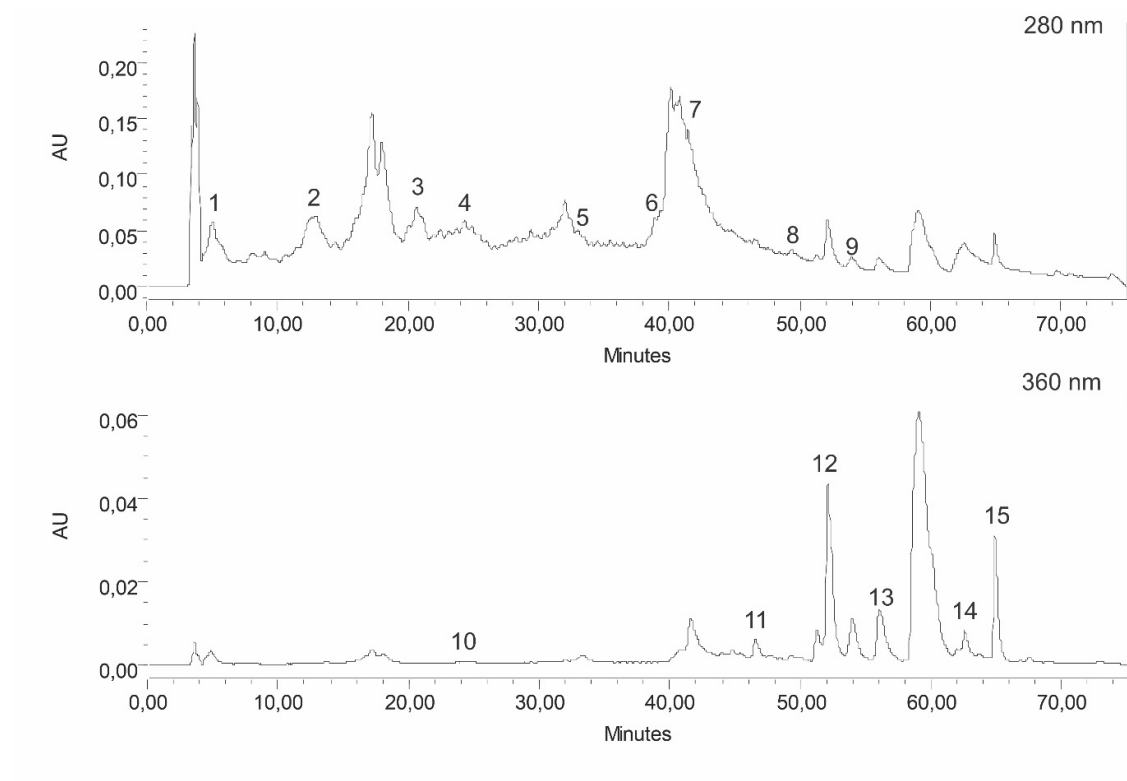

Supplement: Supplementary file 1 [file molecules-31-01643-s001.zip › Figures S2.pdf]

Abundance

TC:extrac LD\data.ms

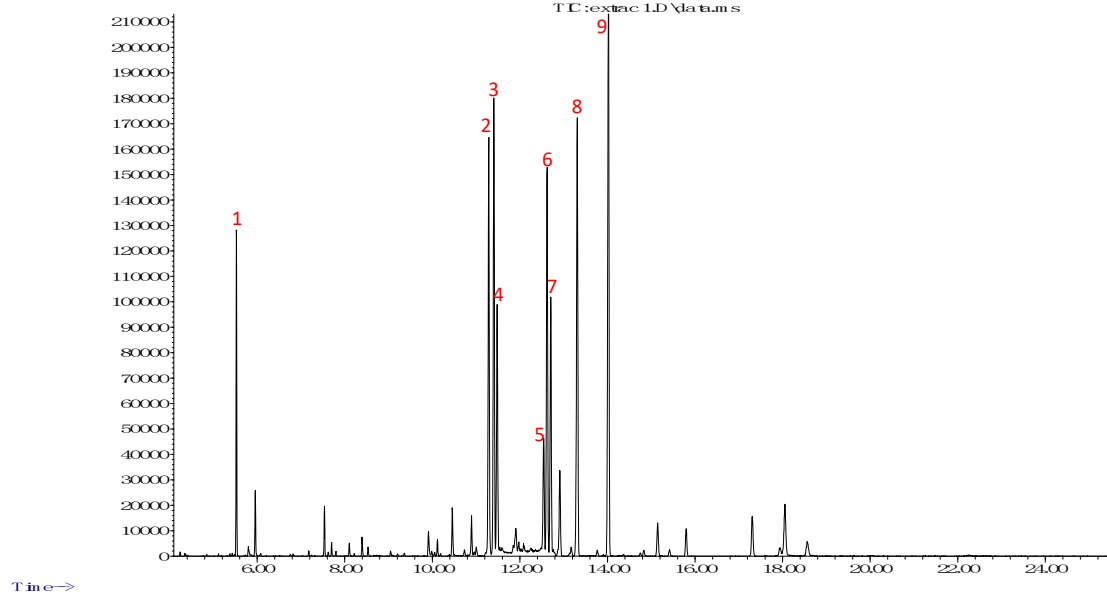

Time→

Supplement: Supplementary file 1 [file molecules-31-01643-s001.zip › Figures S3.pdf]

Abundance

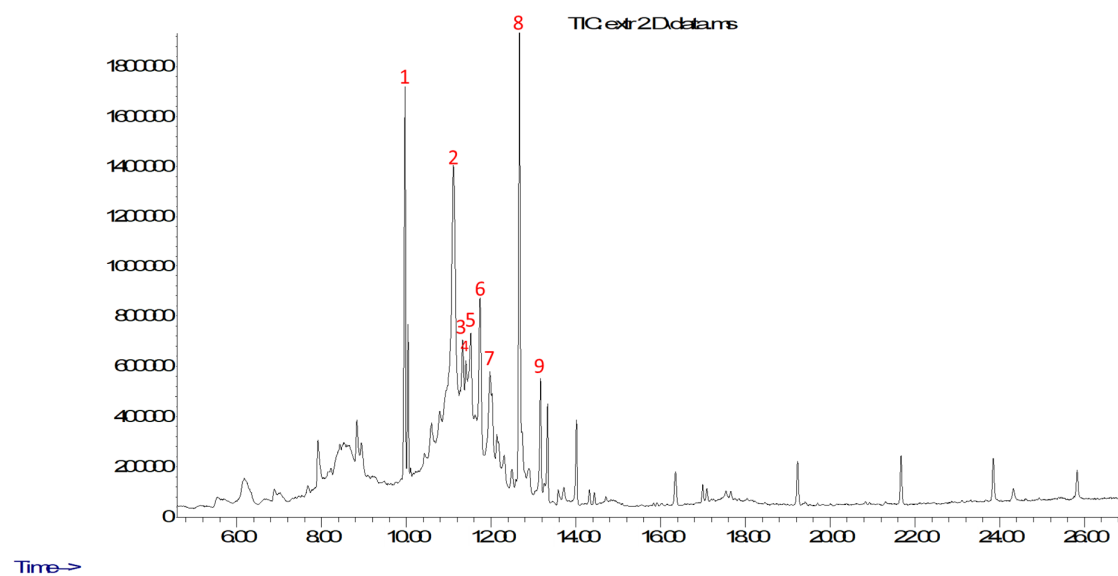

Supplement: Supplementary file 1 [file molecules-31-01643-s001.zip › Figures S4.pdf]
